# Developing Criteria for an Emerging Land Use - *Sphagnum* Moss Harvesting - Through Stakeholder Engagement and Consequent Potential *Sphagnum* Harvesting Area in Finland

**DOI:** 10.1007/s00267-025-02279-y

**Published:** 2025-09-30

**Authors:** Oona Allonen, Maija Lampela, Jukka Turunen, Elina Heininen, Anna M. Laine

**Affiliations:** 1 Geological Survey of Finland GTK, Espoo, Finland; 2 School of Forest Sciences, University of Eastern Finland, Joensuu, Finland

**Keywords:** *Sphagnum* harvesting, land use policy, growing media, spatial analysis, peat mosses

## Abstract

*Sphagnum* mosses are a dominant plant group in boreal and temperate peatlands, significantly contributing to peat accumulation and, consequently, terrestrial carbon stock. *Sphagnum* moss as a potentially renewable alternative for peat is an emerging raw material in the horticultural growing medium industry – hence *Sphagnum* is harvested or farmed in multiple countries worldwide. In Finland, *Sphagnum* harvesting is a new land use of peatlands, currently conducted on a small scale, preferentially on sites previously affected by other types of land use. However, with ample suitable harvesting areas available, such as forestry drained peatlands with low timber production, *Sphagnum* harvesting has the potential to become a significant land use practice. To assess the available *Sphagnum* harvesting land resources in Finland, we employed collaborative working methods, including repeated workshops with stakeholders and semi-structured interviews to establish criteria for site selection. Through stakeholder engagement, the criteria were selected, further modified, and used in spatial analysis to delineate potential harvesting areas and estimate land area. The criteria formulation involved several stages, including identifying existing land-use restrictions and other possible constraints on peatlands, and assessing where suitable *Sphagnum* yield and production costs can be achieved. The resulting area estimate ─ 241,000 hectares of potential *Sphagnum* harvesting area ─ is substantial, accounting for up to 3% of all peatland areas in Finland. It also exceeds the estimated area needed for *Sphagnum* harvesting to replace peat as a growing medium in Finland. The stakeholder engagement process revealed the need for further regulation of *Sphagnum* harvesting if the activity is upscaled.

## Introduction

Northern peatlands play a crucial role in terrestrial carbon (C) sequestration, storing an estimated 400–500 Pg C as peat (Loisel et al. 2014), which accounts for nearly 50% of the atmospheric CO_2_ stock (Le Quéré et al. 2018). *Sphagnum* mosses, a dominant plant group in boreal and temperate peatlands, are key contributors to peat accumulation (Clymo and Hayward 1982; Van Breemen 1995). Beyond their ecological role, *Sphagnum* mosses possess exceptional physical and chemical properties, such as high water retention capacity, antimicrobial activity, pH regulation, and tolerance to desiccation, making them an ideal candidate for use as a horticultural growing medium (Taskila et al. 2016, McKeon-Bennett and Hodkinson 2021, Guêné-Nanchen and St-Hilaire 2022, Müller and Glatzel 2021).

Globally, of the total of 67 million m^3^ (M m^3^) of growing media used annually, approximately 40 M m^3^ consists of peat (Blok et al. 2021). In Finnish greenhouse cultivation, peat is the most used growing medium, accounting for up to 90% of the used growing media (Silvan et al. 2019). In Finland in the year 2021, the extraction for horticultural, bedding and environmental peat was 3.5 M m^3^ (Natural resources institute Finland 2021) of which an estimated 1–2 M m^3^ is used for growing media purposes (Silvan et al. 2019, Heiskanen 2021), while 2.7 M m^3^ of peat was extracted for energy (Natural resources institute Finland 2021) with declining trend. The use of peat has become increasingly less attractive due to its significant environmental impacts, including greenhouse gas emissions and the detrimental effects of peat extraction on mire ecosystems and biodiversity.

One option to replace peat in growing media uses is *Sphagnum* moss. If *Sphagnum* regrowth can be ensured, *Sphagnum* moss can be considered more sustainable than peat (McKeon-Bennett et al. 2021, Müller and Glatzel 2021). Gaudig et al. (2018) distinguish two different paths for *Sphagnum* harvesting: *Sphagnum* gathering, i.e., collecting from wild populations that are not managed or minimally managed and *Sphagnum* farming, i.e., cultivating *Sphagnum* biomass for harvesting. *Sphagnum* gathering is done in some scale, e.g., in Chile and Finland and *Sphagnum* farming trials have been conducted in, e.g., Canada, Germany, The Netherlands, Latvia, China and Japan (Gaudig et al. 2018, Gao et al. 2023, Pouliot et al. 2015, Hoshi 2017). There are also ongoing trials in Finland in an old peat extraction area in South Ostrobothnia region and in Eastern Finland near Joensuu. The area used for *Sphagnum* harvesting is still minimal if compared to the potential need for replacing peat in growing media (Hirschler et al. 2022). In Finland, the area for *Sphagnum* harvesting is currently 224 ha on 31 separate sites (Geological Survey of Finland 2025) and there is no statistics on the harvested volumes.

As *Sphagnum* harvesting removes the carbon sequestered to the living vegetation and prevents peat accumulation, it is essential to ensure *Sphagnum* renewal to consider *Sphagnum* harvesting a sustainable practice. Site selection should target areas where suitable water table levels can be achieved, where nutrient levels are low, and active measures, such as moss layer transfer by spreading *Sphagnum* moss fragments (described in Quinty and Rochefort 2003) and water table management, should be implemented after harvesting. Harvesting should also be targeted to already degraded sites such as unproductive forestry drained areas, where mire ecosystem is degraded but the tree growth is not sufficient for commercial harvesting. To target the harvesting to the living moss and to prevent problems related to high water levels afterwards, use of low-disturbance machinery and a moderate harvesting depth not exceeding 25 cm is recommended (Myllyviita et al., preprint 2025). When regrowth is ensured, transitioning to *Sphagnum* moss harvesting in a cyclical and renewable basis (i.e., farming, on forestry drained peatlands or other degraded peat areas) could help mitigate the climate impact of growing media production (Gaudig et al. 2018). There are not yet results from long-term trials on *Sphagnum* farming in Finland, thus the measures for sustainable production are based on experience from other countries. There is no consensus on the optimal rotation time for *Sphagnum* farming in Finland, but an assumption based on earlier experience has been between 20–30 years (see Silvan et al. 2019). Based on the research carried on harvested sites in Finland, greenhouse gas emissions are dependent on the water table and vegetation succession. Harvesting generally lowers the soil surface which may lead to high water tables and pooling of water before vegetation growth compensates the lowering. Karjalainen et al. (2025) describe how CO_2_ uptake may temporarily increase in harvesting sites, but at the same time, methane emissions are elevated, especially on wet sites. On the study sites described in Karjalainen et al. (2025), it took on average 5–7 years after harvesting for the GHG balance to resemble that of an unharvested control site. Life cycle analysis of *Sphagnum* moss growing medium (Myllyviita et al., preprint 2025) shows that when renewal of *Sphagnum* is successful and site conditions favorable, *Sphagnum* moss can achieve a negative carbon footprint. There are also impacts on water quality from *Sphagnum* harvesting: underlying peat soil may become subject to erosion, which in turn may increase the load of organic matter, solids, total phosphorus, and nitrogen to the watercourses (Laine-Petäjäkangas et al. 2024).

Finland is divided into two main peatland regions: raised bogs in the western and southern parts of the country and aapa mires in the northern and eastern parts of the country. Currently, the majority of the *Sphagnum* harvesting activity has occurred in the raised bog region, where nutrient-poor mire site types and *Sphagnum* species commonly regarded as most suited (species from subgenera *Acutifolia* and *Sphagnum*) for growing media prevail. Nevertheless, based on the information provided by the producers, there is a wide variety of species that can be used in growing media products. Thus, the peatland region or species may not fully limit the harvesting activity in different parts of the country.

It has been estimated that replacing peat entirely with *Sphagnum* biomass as a growing medium in Finland would require approximately 60,000 ha of land for *Sphagnum* harvesting (Silvan et al. 2019). This estimate is based on an annual need to replace 2 million m^3^ of extracted peat, an average *Sphagnum* moss harvest yield of around 1000 m^3^/ha, and a 30-year harvesting cycle, after which the same area could be harvested again.

Due to the fairly recent start of the *Sphagnum* harvesting activity, there is no direct legislation or regulation for *Sphagnum* harvesting in Finland. However, Laakso and Heinilä (2023) highlight several existing Acts that are applicable to *Sphagnum* harvesting, including the Forest Act, Nature Conservation Act, Environmental Protection Act, and Water Act. They also point out the need for clearer regulatory measures, potentially through the Environmental Protection Act (527/2014), or the development of soil legislation. In practice, non-binding guidelines influence peatland land use, such as the National Peatland Strategy (Finnish Government 2012), which restricts land-use changes to peatlands that have already been impacted by drainage and have lost their natural state. Additionally, a committee has been established to address regulatory concerns and provide guidelines for *Sphagnum* harvesting (Ministry of the Environment 2022). Overall, the lack of clear guidelines has caused concerns among stakeholders (SLL 2020a; SLL 2020b; SLL 2022), and the need to develop regulatory framework before the sector expands further is widely recognized (Ministry of the Environment 2022).

The aims of this study were to develop criteria for identifying suitable *Sphagnum* harvesting areas and to assess the available land resources in Finland based on these criteria. We concentrate on the *Sphagnum* harvesting in the manner of gathering from degraded sites not necessarily prepared for harvesting activity. To reach broad acceptance and recognition, the criteria were defined in collaboration with relevant stakeholders. We then applied these criteria to estimate the total land area suitable for *Sphagnum* harvesting and examined how variations in the criteria impact the resulting land area distribution across Finland.

## Material and methods

### Defining Criteria for Recognizing Potential *Sphagnum* Harvesting Sites

**Fig. 1:**
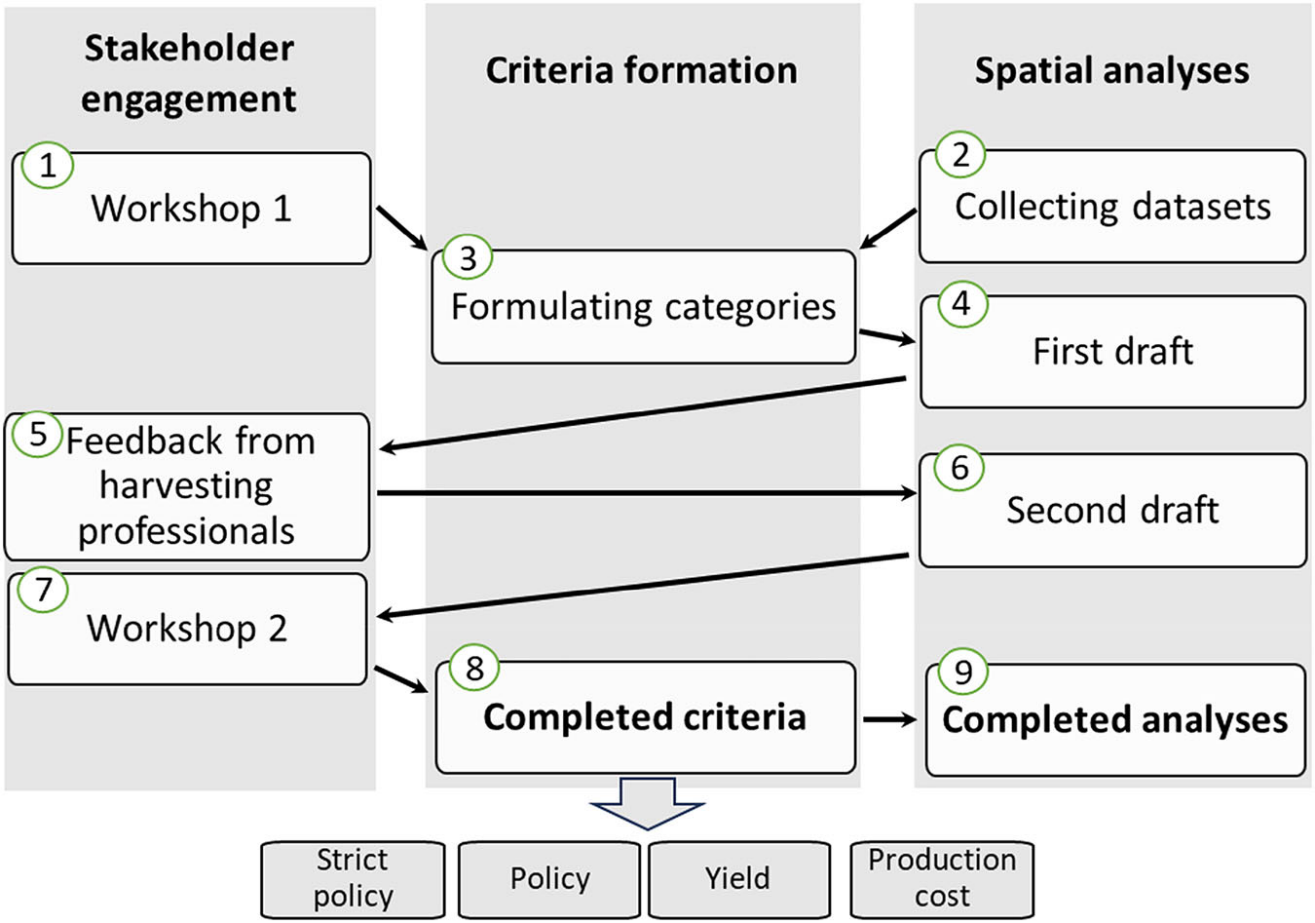
Schematic presentation of the workflow for the study. Steps 1–9 are numbered. Stakeholders participating in workshops comprised environmental NGO and organizations, authorities, harvesting industry representatives and researchers

As a baseline for forming the criteria for *Sphagnum* harvesting, we used the work carried out by the committee set by the Finnish Ministry of the Environment to address the questions and set guidelines to *Sphagnum* harvesting (Ministry of the Environment 2022). Our work consisted of three parts: stakeholder engagement, criteria formation and spatial analysis (Fig. 1), described in detail below.

#### The first workshop

The work was initiated by holding a workshop in November 2022 (Fig. 1, step 1) for stakeholders including environmental NGO and organizations (the Finnish Association for Nature Conservation), authorities (Ministry of Agriculture and Forestry of Finland, Ministry of the Environment, ELY Centre), harvesting industry representatives and researchers (University of Eastern Finland, Natural Resources Institute Finland, Geological Survey of Finland) to discuss *Sphagnum* harvesting and farming in Finland and relevant criteria that should either limit or steer *Sphagnum* harvesting site selection. The workshop applied semi-structured stakeholder discussions in groups of 5–7 people with a facilitator, to record and evaluate the different values and views on the topic. The discussion topics in the workshop were ecological, technical and production cost-related criteria for *Sphagnum* harvesting site selection, renewal of *Sphagnum* in harvested areas and *Sphagnum* farming. Based on the results of the workshop, we categorized the criteria to have a basis in **Strict policy and natural state classification, Policy, Yield** and **Production cost** (Fig. 1, step 3).

##### Strict policy and natural state

The “Natural state” classification on Finnish peatlands (in peatland basin level, area of 5 ha or more) was created by the Geological Survey of Finland to be applied in land use planning. The scale is from 0 to 5, 0 being the most degraded and 5 being pristine. In natural state 0, draining has altered the hydrology irreversibly, and vegetation has changed drastically. The water table has lowered thoroughly in the peatland basin. In natural state 1, draining has altered the water regime thoroughly, and changes in vegetation are distinct. The water table has lowered thoroughly in the peatland basin. In natural state 2, undrained parts remain in the peatland basin, but drainage prevents hydrological connections between the peatland and surrounding mineral soil areas. For raised bogs, drainage has affected the water regime of the center of the bog and the sloping parts around it. Drainage partially affects the areas where there are no ditches as well. Typical mire vegetation has been altered: for example, increased dwarf shrubs and saplings. In natural state 3, most of the peatland basin is undrained. On aapa mires, drainage doesn’t completely prevent water from entering from surrounding areas and doesn’t cause significant drying further away from ditches. On raised bogs, ditching can affect lagg areas (for more detailed description, see Toivonen et al. 2022). The natural state of a peatland describes the drainage status of a peatland basin and how it affects the hydrology and vegetation. As the degree of drainage and its effect on hydrology and vegetation have a gradual variation, the automatically outlined natural state for a peatland basin is always an approximation and often a compromise between two classes (Toivonen et al. 2022). The Finnish National Peatland strategy (Finnish Government 2012) recommends that land use change should take place only on peatlands of the lowest natural states: 0 and 1, 2 with discretion (considering regional aspects, see Ministry of the Environment 2015) and 3 in exceptional cases. In workshops, it was agreed that the natural state classification should be used to exclude the most pristine peatlands from *Sphagnum* harvesting land use, thus only peatlands with a natural state of 0–2 were included in the spatial analysis.

In addition, we investigated strict policies and laws that limit land use on peatlands as no-go areas for *Sphagnum* harvesting, namely state-owned protected areas, privately owned protected areas, Natura 2000-areas, Wilderness reserves, the especially important habitats protected under the Forest Act and archeological cultural environment heritage sites.

From peat extraction regulation, we decided to adopt a criterion to not locate *Sphagnum* harvesting sites on important groundwater bodies to not compromise aquifer water condition (Water Act, VL 587/2011, Väyrynen et al. 2008). Similar to peat extraction also this land use could potentially affect the water table and run-off leading to degradation of a groundwater body. As an example, we considered surroundings (30 m) of natural springs and groundwater bodies that are important water sources or connected to ecosystems dependent on groundwater supply, such as spring-fed fens and spring-fed ponds (Britschgi et al. 2018) unsuitable for *Sphagnum* moss harvesting.

##### Policy

In the workshop, valuable areas not protected by law but worth keeping out of *Sphagnum* harvesting activities were recognized. Based on discussions, areas proposed for conservation in the Complementary mire conservation program (Alanen and Aapala 2015) were considered ecologically too valuable for *Sphagnum* harvesting. These areas have been recognized to have particularly high nature values or importance for enhancing connectivity of protected mire sites (Aapala et al. 2021).

As stated before, the natural state class of a peatland is defined for a whole peatland basin. However, the drainage situation can differ drastically within one peatland basin (Toivonen et al. 2022). Based on the workshop, it was considered important to ensure that *Sphagnum* harvesting should focus on areas truly influenced by previous drainage, and not on the sections of peat basins that still support rather intact peatland vegetation and functions. To facilitate this, we adopted a distance of 50 m from drainage from Sallinen et al. (2019) to describe and target the peatland area that is influenced by a ditch.

##### Yield

In the yield category, we defined factors that affect the amount of *Sphagnum* moss to be retrieved. *Sphagnum* harvesting industry representatives contributed to this criteria category in particular. We ruled out areas that lack *Sphagnum* cover for technical reasons: namely, peat extraction sites and agricultural peat fields as these are included in peatland data within a natural state of 0. The potential for *Sphagnum* harvesting has been recognized in forestry drained peatlands. Our aim was to target areas that likely have a *Sphagnum* cover or areas that would be suitable for reinstating *Sphagnum* moss growth before *Sphagnum* harvesting, and where renewal after harvesting could be ensured. We aimed to target the nutrient-poorer oligo- and ombrotrophic, originally treeless peatland types, where *Sphagnum* growth has proved to be greater after restoration, as opposed to more nutrient-rich meso- and eutrophic forested peatland types (Laatikainen et al. 2025). As we did not have reliable enough spatial data on mire site types for all Finnish peatlands, we used tree volume of a maximum of 30 m^3^/ha as a criterion, to indirectly target these nutrient-poor peatland types. Thresholds of ≤10 and ≤50 m^3^/ha were also discussed and implemented in spatial analysis. Low density of tree stand on drained peatland can be seen as an indication of lower nutrient levels, and *Sphagnum* mosses are naturally more abundant in nutrient-poor (poor fen, bog, pine mire) than in nutrient-rich peatlands (fens and spruce mires). Low density of the tree stand can also indicate poor success of forestry draining, meaning a higher water table level that is more favorable for Sphagnum mosses. These open areas are prone to more light as well, which can favor *Sphagnum* mosses. 30 m^3^/ha has been used as a threshold for low tree production on drained peatlands (Laiho et al. 2016). Low volume of the tree stand indicates poor tree growth and suggests that drainage has not been successful and therefore water table could still be somewhat suitable for *Sphagnum* moss growth.

##### Production cost

Production cost was recognized to affect the suitability of an area for commercial *Sphagnum* harvesting. Location is key in the regional scale (vicinity of growth medium processing plants) and local scale (access to the area by road). We formed criteria based on the local scale and did not consider processing plant distances. In the first draft for spatial analysis, we used 500 m as a maximum distance from the edge of the harvesting site to the road, but also considered distances of 300 m and 100 m. The size of the area also contributes to the practical usefulness of a harvesting area. Industry representatives considered 5 ha minimum area for a harvesting site. If multiple, potential harvesting sites were closely situated (700 m or less), a minimum area per site was set to 0.5 ha.

#### Spatial analysis drafts and feedback

While forming criteria, we emphasized factors that would be useful in spatial analysis – one requirement being that there had to be available spatial data on the matter. Based on discussions in the first workshop, suitable datasets were acquired and the first draft for spatial analysis was done (Fig. 1, steps 2 and 4) using the criteria described above. The analysis was done using ArcGIS Pro 3.2.2. software. Processing details and datasets are described in “Spatial analysis to define the potential *Sphagnum* harvesting area in Finland”.

**Fig. 2:**
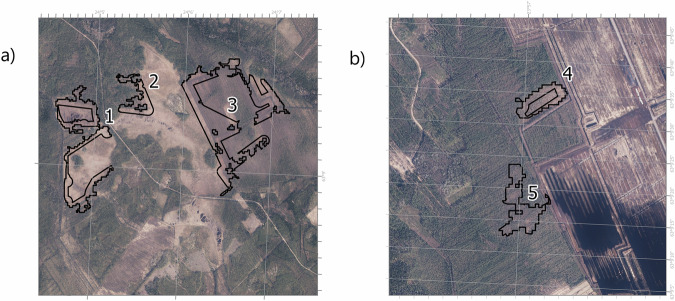
An example of potential harvesting area outlined in the first spatial analysis. These areas were evaluated by the *Sphagnum* harvesting companies. **a** Polygons 1, 2 and 3 around an open mire, limiting to 50 m vicinity of surrounding ditches. On a scale from 0 to 5, these polygons were scored at 3 and 4. Lower scores resulted from concerns on accessing the area, uncertainty of the amount of available *Sphagnum* resource (on polygon number 3) and being partly too wet (on polygon number 1). **b** Polygons 4 and 5 next to a peat extraction area. These polygons were scored at 4 and 5

During the criteria forming process, we asked professionals from two *Sphagnum* harvesting companies to evaluate the areas that resulted from this first draft for spatial analysis (Fig. 1, step 5). We sent both parties a random sample of 30 areas for evaluation from different municipalities, both in South Ostrobothnia, and held a semi-structured interview to discuss the suitability of the sample areas for *Sphagnum* harvesting (Fig. 2). We asked the people responsible for the harvesting planning to evaluate the suitability of the area for *Sphagnum* harvesting on a scale from 1–5 (1 being not suitable and 5 excellent suitability) and to give other feedback, if any. We instructed them to use all data that they would normally use when evaluating *Sphagnum* harvesting areas but asked them not to consider the effect of landownership. The evaluation was done without field visits, based on available data and worker’s knowledge on local conditions and prerequisites for the business.

*Sphagnum* harvesting companies gave us feedback on area size: small areas were deemed unprofitable. In both municipalities, almost half (15 and 14) of the 30 sample areas were between 0.5–2 ha, as 0.5 ha was set as the minimum area size in the first workshop. We also got feedback on accessing the area: some sample areas were considered too far from the roads, as the maximum distance to the road was set to 500 m based on the first workshop. We got criticism on some of the areas having too much tree cover and hence expected poor yield. We also got feedback on areas being too narrow strips, following the margin ditches of peatlands.

Some of the sample areas (6 out of 60) were deemed as areas with excellent suitability, some even being areas that were already in plans or in use for *Sphagnum* harvesting (5/60). Most of the areas were deemed unsuitable, with mean score from one municipality being 1.53 and another municipality 2.86. If the areas that got feedback based on small size, accessibility by road or too high tree volume are not counted, as these parameters could be amended, mean scores would be 2.86 (*n* = 7) and 4 (*n* = 11), respectively. When looking at areas that would be chosen with completed criteria (changing area size and distance to road), mean scores are 2.1 and 3.55, respectively.

Based on the feedback, we decided to adjust the following parameters for the second draft of spatial analysis (Fig. 1, step 6): the minimum size of areas that are closely situated was increased from 0.5 to 2 ha and maximum distance to the road was decreased from 500 m to 300 m.

#### Second workshop and completed criteria

The resulting areas from the second draft of spatial analysis and main topics in *Sphagnum* harvesting companies’ feedback were presented in the second workshop in April 2024 organized online for the stakeholders using similar group discussions and facilitators as in the first workshop (Fig. 1, step 7). The stakeholder groups invited were the same as in the first workshop, and there were only slight changes in the attendees: this time, the ministry representatives were not present and researchers from the Finnish Environment Institute attended. Discussion topics were associated with criteria, as opposed to a more general *Sphagnum* harvesting theme in the first workshop. Criteria within each category were discussed in groups. Based on the discussions, some modifications to the criteria thresholds were made: for example, distance to drainage was increased from 50 m to 100 m. This completed the criteria formation process (Fig. 1, step 8). At the end of the workshop, consensus on the final criteria was measured on a scale from “not satisfied” to “satisfied”. All the participants were at least on average satisfied with the outcome.

### Spatial analysis to define the potential *Sphagnum* harvesting area in Finland

After completing the criteria formation, the final spatial analysis was executed (Fig. 1, step 9). The spatial analysis began with vector data on peatlands of natural states of 0–5. We used automatically delineated peatland polygons from Toivonen et al. (2022) that cover peatlands of 5 ha and larger in Finland, including information on natural state classes, and selected those belonging to classes 0–2. We report the area of potential harvesting sites based on different criteria separately for each of these natural state classes. We do this because the workshops did not reach a full consensus on which of these natural state classes to aim for. In addition, measures preceding *Sphagnum* harvesting differ based on how degraded the harvesting site is.

From these polygons, we erased areas that were excluded through the Strict policy category: Natura 2000 -areas and other protected areas, Forest act habitats, groundwater bodies classified as E, 1, I, 1E and 2E, springs with a 30-m buffer zone, and archeological cultural environment heritage sites with a 10-m buffer zone. Spatial data on Natura 2000 areas, other protected areas and groundwater bodies were acquired from the Finnish Environmental Institute (2023a, b, c). Spatial data on habitats protected under the Forest Act was acquired from the Forest Centre (2022), data on archaeological cultural environment heritage sites from Finnish Heritage Agency (2023) and data for springs from the Topographic Database of the National Land Survey of Finland (2023).

To consider criteria from Policy category, we erased Complementary mire conservation program proposed areas based on spatial data acquired from the Finnish Environmental Institute (2023d). In addition, we included only areas that were within 100 m from ditches. To do this, we first retrieved vector data on ditches from the Topographic Database of the National Land Survey of Finland and made a 100-m buffer polygon of ditches. We then only considered potential areas that intersected with the ditch buffer zone.

Criteria in the Yield category were considered in spatial analysis by selecting only areas with low tree volume. For tree volumes, we used MS-NFI (Multi-Source National Forest Inventory) raster maps from years 2015 and 2021 (Natural Resources Institute Finland 2023). MS-NFI maps describe the forest parameters in Finland as thematic maps with a resolution of 16 × 16 m. MS-NFI maps are a result of combining field data from NFI field plots, satellite imagery, digital map data, and other available georeferenced data (Mäkisara et al. 2022). Areas low in tree volume due to harvesting between the years 2015 and 2021 were ruled out using raster calculation: If tree volume decreased more than 30 m^3^ between the years, it was considered a tree harvesting area and excluded from further spatial analysis. Raster cells with tree volume under 11, 31 and 51 m^3^/ha were converted into polygons. We used the intersection tool to match areas resulting from the policy category to low tree volume areas. To recognize and exclude peat extraction areas and agricultural peat fields, we used spatial data from the Topographic Database of the National Land Survey of Finland.

Criteria in the production cost category were implemented by calculating area for each resulting polygon. Before this, polygons were merged and dissolved and aggregated with a small margin to make sure all polygons that shared a border or an angle were considered as one. After this, polygons under 2 ha were dismissed. Small polygons (2 to under 5 ha) that are close by were recognized with the aggregate polygons tool (aggregation distance 700 m, minimum size 6 ha, minimum hole size 0.5 ha). Small polygons under 5 ha were discarded if they did not intersect with aggregated polygons. For resulting polygons, the distance to road was calculated from each area margin. Polygons within 300 m from roads were selected as potential harvesting sites. For spatial data on roads, we used Digiroad (road categories 1–7, all suited for forestry logistics) from the Finnish Transport Infrastructure Agency (2023).

To showcase how the potential harvesting area is situated in Finland, Kernel Density (in the Spatial Analysis Tools) was calculated to identify potential *Sphagnum* harvesting areas on a regional scale. Potential harvesting areas were first converted from polygons to points. The raster cell size of 1 km and search radius of 25 km were used in the Kernel Density analysis. Each kernel was weighted for the potential harvesting area size.

## Results

### Criteria

Based on workshops with stakeholders and feedback from *Sphagnum* harvesting companies, a set of criteria was developed to identify areas where harvesting could be feasible (Table 1). The formulation of these criteria involved several stages: first, identifying existing land-use restrictions on peatlands (i.e., strict policy and natural state classification); second, determining additional constrains (policy); where suitable areas for harvesting are available or have the potential for establishing new *Sphagnum* moss cover through hydrological restoration before harvesting (yield); and finally, assessing which of these areas could be applicable for commercial use (production cost) (Fig. 1).

**Table 1:** Completed criteria as a result of Criteria formation through stakeholder engagement (Step 8 in Fig. 1)

	Included	Excluded
Strict policy and natural state classification	Natural states 0–2	Natural states 3–5
		Protected areas: state owned and private
		Natura 2000 -areas
		Wilderness reserves
		Habitats protected under the Forest Act
		Groundwater bodies that are important water sources or connected to ecosystems dependent on ground water supply
		Natural springs (30 m buffer zone)
		Archeological cultural environment heritage sites (10 m buffer zone)
Policy	Near ditches (≤100 m)	Distance to ditches (>100 m)
		Complementary mire conservation program areas
Yield	Tree volume ≤ 30 m^3^/ha	Tree volume > 30 m^3^/ha
		Agricultural peat fields
		Peat extraction sites
Production cost	Near roads (≤300 m)	Distance to roads (>300 m)
	Area size > 5 (isolated areas) OR > 2–5 ha (areas connected or of < 700 m distance to others)	

Criteria are presented based on whether they include or exclude areas based on the criterion. Criteria categories are *Strict policy and natural state classification*, *Policy*, *Yield* and *Production cost*. These criteria were used for completed spatial analysis (Step 9 in Fig. 1)

### Land area suitable for *Sphagnum* harvesting

Of Finland’s total peatland area, which covers 9.1 million ha (Mha) (Turunen and Valpola 2020), 6.2 Mha of peatland basins >5 ha have been delineated and classified based on their natural states (Toivonen et al. 2022). This classified area was used as the total peatland area considered in our study (Table 2). Within this, 3.6 Mha falls under natural state classes 0–2. Based on strict policy and policy criteria, 3.1 Mha of these peatlands could potentially be used for *Sphagnum* harvesting (Table 2). However, when considering moss yield criteria, 233,000 ha are excluded due to their classification as agricultural land or peat extraction site (see “Defining criteria for recognizing potential *Sphagnum* harvesting sites”). Furthermore, applying the tree stand volume criteria within the yield category (≤30 m^3^/ha) significantly decreases the potential area to 416,000 ha. When additional production cost criteria—such as the maximum allowable distance between harvesting areas and the nearest road, as well as the minimum viable harvesting area size—are factored in, the total suitable area for *Sphagnum* harvesting in Finland is reduced to 241,000 ha. The delineations of the resulting areas are publicly available in the Hakku -geological data product service of the Geological Survey of Finland (2024). While initially more hectares fall within natural state classes 0 or 1 compared to class 2, the final assessment—accounting for potential harvesting yield—indicates that a greater portion of the suitable areas are located in peatlands classified as natural state 2 rather than 0 or 1 (Table 2).

**Table 2:**
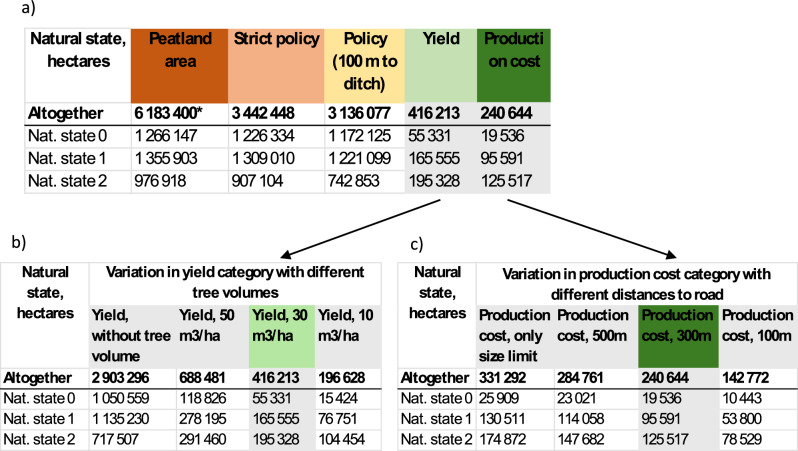
**a** Potential *Sphagnum* harvesting area resulting from spatial analysis

The final result, after considering all restricting factors, is presented in the Production cost column. The progressive impact of each criteria category on the total land area is shown from left to right. The effect of changes in thresholds in the Yield and Production cost categories are presented in tables **b** and **c**. The effect of change in Policy category (with 50 m distance to ditch) and its consecutive effect on the Yield and Production cost is shown in Table S1 in the Supplementary material. *The total peatland area (6183,400 ha) also contains the natural states 3, 4, and 5 (854,268 ha, 798,749 ha, and 931,415 ha, respectively)

**Fig. 3:**
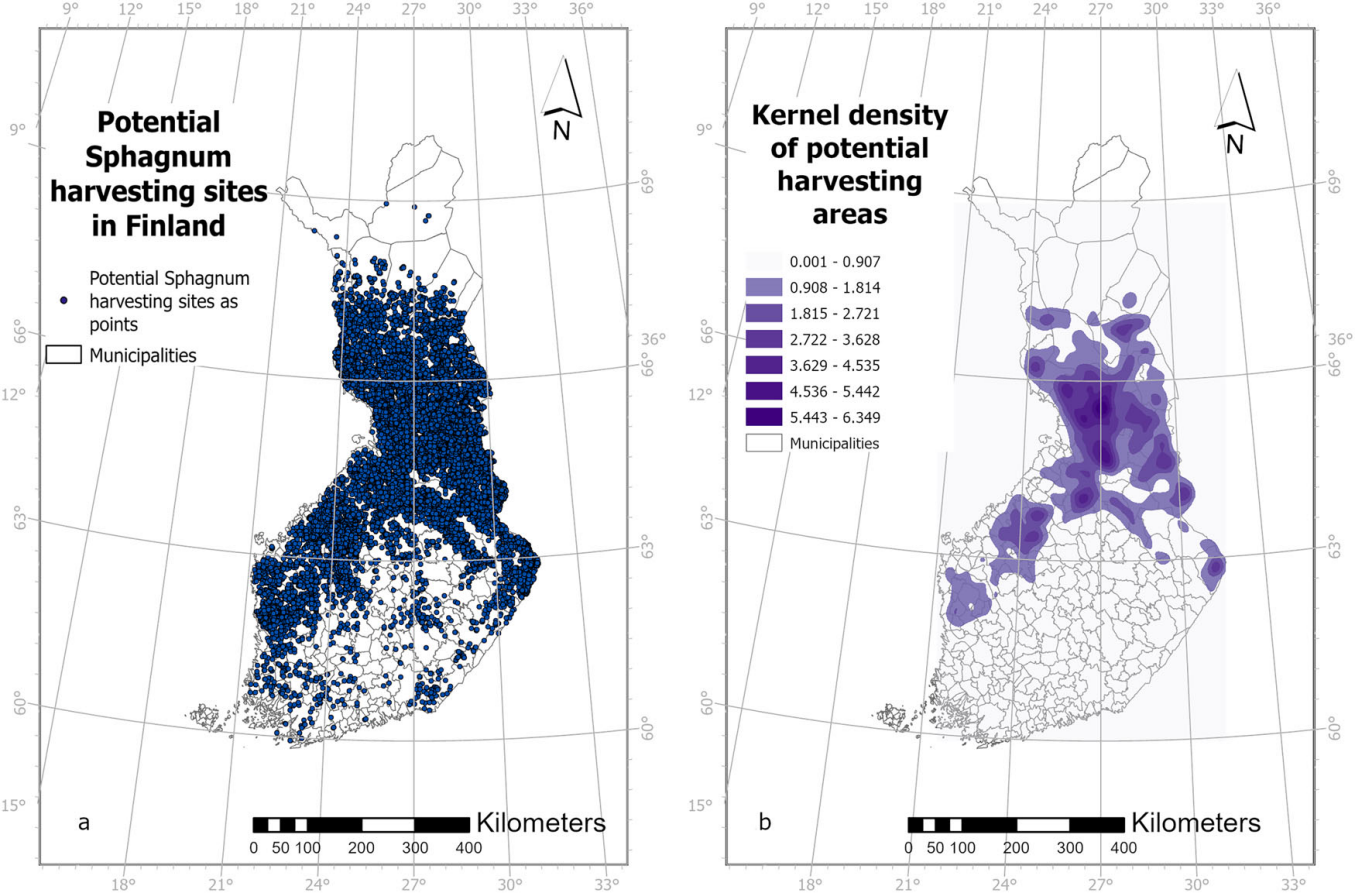
Potential *Sphagnum* harvesting sites in Finland. Sites are shown as **a** points and **b** Kernel density (equal interval) of potential harvesting areas. The color illustrates the relative density, not the specific units

While potential harvesting areas were recognized from each region in Finland (Fig. 3a), most of the potential harvesting areas are situated in Northern Ostrobothnia, Kainuu, the southern parts of Lapland, and along the western coast of Finland (Fig. 3b). Municipalities with some of the highest Kernel Density values are Pudasjärvi, Utajärvi, Vaala, and Ranua.

### Impacts of alternative criteria on potential harvesting area

We examined how different parameters for some criteria affect the resulting harvesting area. In the strict policy category, no compromises were made as all excluded areas were considered no-go areas, but the impacts of choosing stricter or looser policy on natural states is presented in Table 2.

In the policy category, the effect of using stricter policy on impact of ditches, namely reducing the distance to the nearest ditches to 50 m compared to 100 m was examined. Choosing the stricter policy of 50 m reduced the potential harvesting area by ~100,000 ha, leading to 143,000 ha potential harvesting area (see the effect of all changes in thresholds when the distance to ditch is 50 m in Supplementary material in Table S1).

**Fig. 4:**
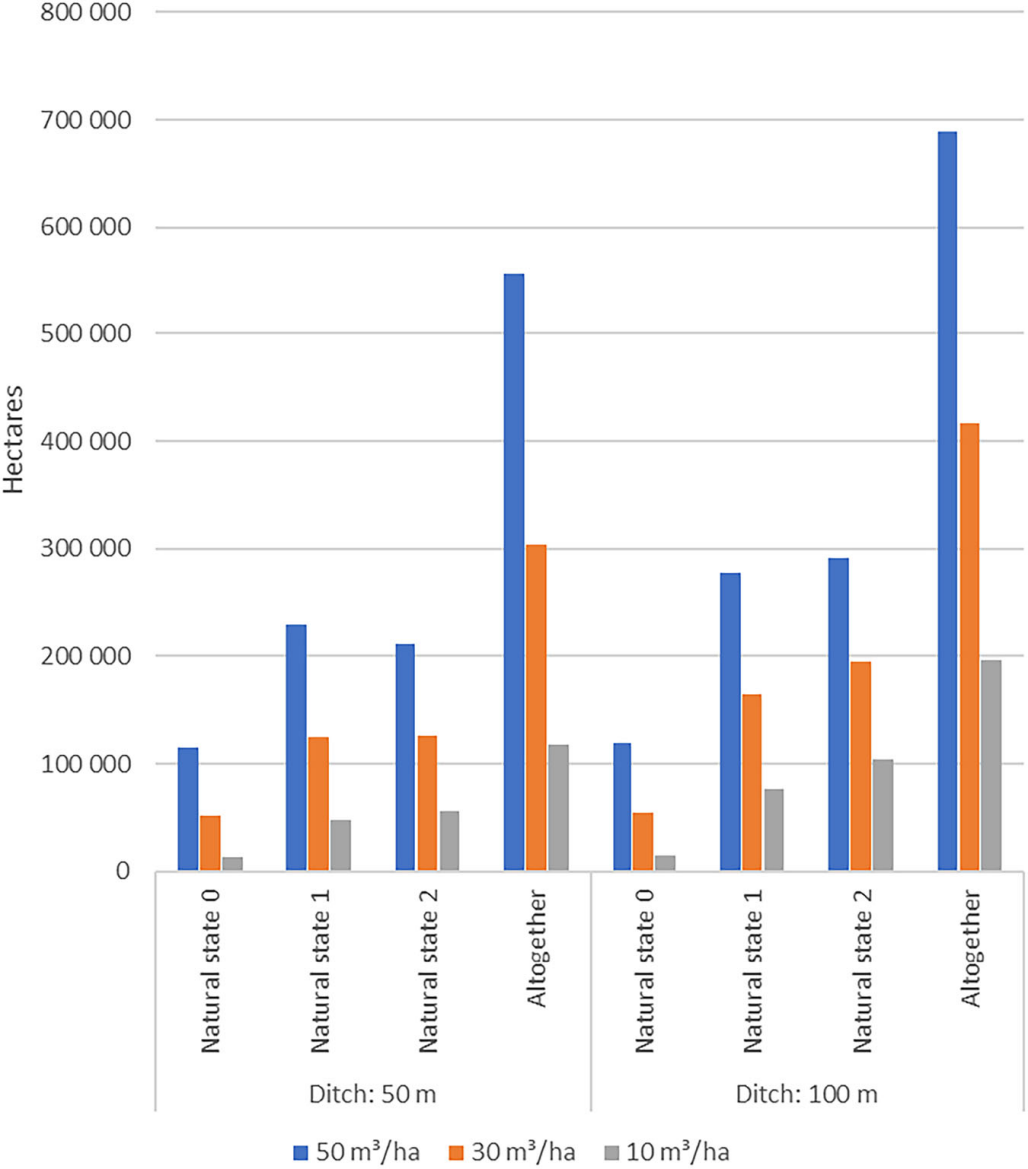
Hectares (ha) in Yield category calculated with different tree volume thresholds

In Yield category, the effects of different tree volume (≤10, ≤30 and ≤50 m^3^/ha) thresholds as a criterion were compared (Table 2b). The tree volume has drastic impact in harvesting area. Compared to 416,000 ha land area based on the ≤30 m^3^/ha threshold, allowing a bigger tree volume, the resulting total land area is bigger, namely 688,000 ha with threshold of ≤50 m^3^/ha. When restricting selection to ≤10 m^3^/ha, the land area is only 197,000 ha (Fig. 4).

In the production cost category, the impacts of different minimum distances to the road were calculated (Table 2c). Distance to road affected the resulting land area as follows: Without considering distance to road at all, potential harvesting area would have been 331,000 ha (area based on yield category limited by the size of single harvesting sites only). Of this, 285,000 ha was within 500 m, 241,000 within 300 m, and 143,000 ha within 100 m from road.

## Discussion

### Stakeholder engagement

The work of formulating the harvesting criteria was built on top of the results of the National peatland strategy (Finnish Government 2012) and the committee that addressed the questions and set guidelines to *Sphagnum* harvesting (Ministry of the Environment 2022). As the subject of *Sphagnum* harvesting was known to have conflicting interests in economic and nature perspectives, stakeholder engagement was seen instrumental to the criteria formulation process. Stakeholder deliberation is promoted for example by EU to mainstream responsible research and innovation (Nielsen et al. 2017) and for effective outcomes, identification of the key stakeholders is considered essential already at the planning phase of the work (Nielsen et al. 2017, reed et al. 2018). We thus paid special attention to finding relevant stakeholders and ensuring their balanced participation in the criteria formulation process. We selected workshops as one working method proven to be well-suited for advancing complex and ill-defined matters (Hermanns et al. 2017, Ørngreen and Levinsen 2017) such as this criteria formation, combining complex set of items related to land use, legislation, policy steering, ecology, economics, and social and practical issues. The first workshop and feedback from harvesting companies in parallel with spatial analysis created material for discussion and decision-making on the criteria for the second workshop. After a wide range of views presented in the first workshop, an acceptable consensus was reached within the second workshop, which implies that the selected working methods were effective in delivering a set of criteria that caters somewhat to the needs of different stakeholders. The considerably long time between the two workshops (1.5 years) may also have contributed to the acceptable results of the second workshop, as the debate around the topic had had time to settle and stakeholders had become more aware of the prerequisites of the *Sphagnum* harvesting activities.

### Criteria for *Sphagnum* harvesting sites

To facilitate future development of binding legislation or other policy steering for *Sphagnum* harvesting, developing site selection criteria and testing the impacts of different criteria parameters on potential harvesting area was seen as elemental. We divided the criteria into four categories to facilitate the process.

Most of the criteria included in **the strict policy** category, largely based on existing legislation (such as protected areas or groundwater bodies), were well accepted by the stakeholders. However, the criterion natural state classifications of peatlands, that was adopted from the National peatland strategy (Finnish Government 2012), where it was at first hand developed for guiding the peat extraction site selection, caused disagreement. The Natural state classification itself is based on a high amount of data from both remotely sensed and field observations with high accuracy. Yet the basis for classification (for example, the definition of a “peatland basin”) and guidelines for its use have been debated. In this case, the stakeholders disagreed on which natural states would be feasible for *Sphagnum* harvesting. Two polar opinions were discussed: *Sphagnum* harvesting should be strictly steered to peatland basins with natural state 0 or 1, that typically do not contain undrained sections i.e., areas at which characteristic peatland structure and functions remain (Toivonen et al. 2022). This raised concerns on how to ensure renewal of *Sphagnum* mosses on such severely hydrologically degraded sites, and it was argued that yield and passive renewal of *Sphagnum* moss are inadequate at such peatlands. Based on this, it was suggested that *Sphagnum* harvesting should be directed to basins with natural states 2 and 3. As the spatial data on natural state classification on all peatland basins in Finland is automatically classified (Toivonen et al. 2022), concerns were raised about its reliability. Especially in category 2, there could be more pristine parts within the peatland basin than per the classification description. Similarly, the use of natural state classification in the context of peat extraction has encountered criticism, especially on how the peatland-basin level classification neglects smaller-scale variation in habitat condition (Herranen 2018). As an outcome, at this study we considered natural states of 0–2 as recommended in the National peatland strategy (Finnish Government Valtioneuvosto 2012) with a presumption that harvesting companies need to be prepared to take active measures to ensure *Sphagnum* moss renewal. This decision was considered somewhat satisfactory by all parties.

To overcome the deficiencies of the natural state classification, and to respond to the need to create sustainable practices, two more criteria were created comprising the **policy category**. Firstly, it was agreed with the stakeholders that harvesting should be limited to the already degraded, drained parts of the peatland basin, despite the natural state class. The surface topography, vegetation, peat properties and amount of water entering the area have severe and site-specific effects on the effectiveness of the drainage in peatlands, but we did not find a way to incorporate these variables to the analysis. We thus used a limit of 50 m from the nearest ditch for classifying areas that are drainage-influenced vs. undrained, based on earlier experiences and generalizations on the reach of drainage impact on peatland vegetation (Sallinen et al. 2019). However, the spatial analysis showed that 50 m distance often led to narrow strips not economically feasible based on the feedback from harvesting companies. Consequently, we changed the limit to 100 m, adopted from Ågren et al. (2024) for classifying areas that are drained vs. undrained based on distance from ditch. This, of course, is also a generalization, and the effect of the drainage varies based on the site properties. Secondly, areas with recognized high natural values belonging to the Complementary mire conservation program proposal were considered not suitable for *Sphagnum* harvesting. Most of the area (79%) in that proposal is, however, situated on peatlands with a natural state higher than 2, thus this criterion seldom further affects harvesting site selection.

While **the yield** category related to the amount of *Sphagnum* material available for harvesting is not essential for legislative or regulative purposes, it greatly affects the economic feasibility of the activity. The amount of *Sphagnum* mosses in drained peatlands is known to vary based on peatland site type, so that the nutrient-poor sites, in Finnish classification, Dwarf shrub type and Cladonia type (Laine et al. 2018), tend to have more abundant *Sphagnum* cover even decades after drainage (Laine et al. 1995, Kokkonen et al. 2019). In addition, the more effective the drainage is, the lower the abundance of *Sphagnum* mosses (Ågren et al. 2024). Based on unpublished data (the Geological Survey of Finland) and discussions with harvesting professionals, the low density of trees that we applied in the spatial analysis of the potentially suitable harvesting area is a relatively good preliminary estimate for high *Sphagnum* yield and therefore for the suitability of harvesting areas. Nevertheless, in practice, a field verification is needed when planning real harvesting activities.

In our spatial analysis, the yield category had the biggest impact on the area estimated suitable for *Sphagnum* harvesting. At the same time this category had the highest uncertainty. We had to rely on the available tree volume data as an indirect indicator of the *Sphagnum* yield, as the spatial data available on peatland site types in Finland (Middleton et al. 2023) was not applicable for detailed examinations.

In the **Production cost** criteria category we particularly emphasized harvesting companies’ input. The most important criteria were the distance to the road and size of harvesting area, for which based on the discussions in the first workshop and on person-to-person discussions with the harvesting company professionals, were given limits 500 m and 0.5 ha, respectively. Nevertheless, only when the areas resulting from the first draft were presented in the real context in the GIS software combined with aerial imagery, the professionals of the harvesting companies could truly evaluate the outcome of these choices. Consequently, this led to changes in the parameters (to 300 m distance to road and 2 ha area size) to better correspond with the current site selection practices and also likely to correspond with the actual potential of sites for *Sphagnum* harvesting.

Another aspect affecting the production costs is the distance to the nearest existing growth medium processing plants. As we aimed at country-wide spatial analysis for potential areas for *Sphagnum* harvesting in this study, such regional aspects were not considered. However, the spatial analysis showed high harvesting potential in the regions of current harvesting activity, namely in Southern and Central Ostrobothnia.

### Potential *Sphagnum* harvesting area in Finland

Our spatial analysis indicates that potential *Sphagnum* harvesting areas are primarily located in Finland’s most peatland-rich regions, as expected. The identified sites vary widely, ranging from severely degraded forestry-drained peatlands to the outskirts of bogs and fens with pristine topographic characteristics, with most sites falling somewhere in between. We estimate that 241,000 ha is suitable for *Sphagnum* harvesting, a substantial area, accounting for up to 3% of all peatland area of Finland. When compared to the estimated land requirement of 60,000 ha needed to replace peat-based growing media in Finland (Silvan et al. 2019), our findings suggest that the potential area (241,000 ha) far exceeds this demand.

### Identified criteria not possible to include into the spatial analyses

Several aspects that influence the implementation on *Sphagnum* harvesting, which could not be conveyed into the spatial analysis, were identified during the criteria development process. Land ownership is a major constraint, as harvesting is only viable on sites where landowners find the transition from forestry to *Sphagnum* cultivation economically appealing. In Finland, fragmented land ownership may further complicate the feasibility of *Sphagnum* harvesting, particularly when hydrologically unified or economically large enough areas are divided among multiple landowners with differing goals. Additionally, some roads providing access to potential harvesting sites are privately owned, which may restrict transportation and logistics.

Species protected under the Nature Conservation Act, Decree, or EU Habitats Directive were not included as criteria in the spatial analysis. This exclusion was due to technical challenges, as some sensitive species data is generalized to protect their locations and is therefore unsuitable for spatial modeling. Consequently, it remains the responsibility of *Sphagnum* harvesting companies to investigate the designated area for protected species before initiating operations.

Another critical factor raised during stakeholder workshops was the renewal potential of *Sphagnum* moss after harvesting, which is essential for the long-term sustainability of the harvesting activity. Ideally, harvesting should be limited to sites where hydrological restoration and techniques such as moss layer transfer (described in Quinty and Rochefort 2003) can effectively support the regeneration of *Sphagnum* moss. Thus, site-specific assessments should always be carried out before harvesting.

## Conclusions

This study presents a structured approach to developing criteria and estimating potential areas for an emerging land use in a complex setting that integrates legal frameworks, policies, views and needs of various stakeholder groups. To address the sometimes conflicting views and interests, we actively involved stakeholders in the criteria development process through workshops and discussions.

Our findings suggest that, given the potential harvesting area identified in our analysis and the growing demand for sustainable growing media, *Sphagnum* harvesting has potential to become a significant land use practice in peatland-rich regions. However, we have identified several challenges in current practices that must be addressed to ensure *Sphagnum* harvesting is truly renewable, as well as both climate-wise and economically sustainable.

During the workshops, renewal of the harvested areas was repeatedly emphasized as the greatest obstacle for sustainability. One solution for this would be *Sphagnum* paludiculture with a cyclic harvesting method that would also partly answer to the needs of climate protection. It would start from rewetting of a degraded peatland, after which *Sphagnum* moss cover is allowed to establish and grow prior to first harvesting, then renewal of *Sphagnum* moss is ensured with practices such as moss layer transfer technique and water table management, and harvesting is repeated in a cycle of 10–30 years depending on *Sphagnum* growth speed. This cyclic production style would, in the long run, reduce the area needed for *Sphagnum* harvesting, and it would ensure that *Sphagnum* mosses and peatland functions are re-established after harvesting. In addition, it could offer a commercially attractive way to improve the hydrology of unproductive drained peatlands, a measure that Turunen and Valpola (2020) point out as one of the key measures to sustainable carbon management of peatlands. Moreover, *Sphagnum* paludiculture on cut-over peatlands and former peat fields should be promoted.

The estimated potential harvesting area of 241,000 ha should not be viewed as a fixed value but rather an example of the opportunities available based on specific methodological choices made in a stepwise process. It serves as an informative example of the potential within Finland and provides a foundation for policy development and land use planning. Despite the inherent uncertainties, the estimated area highlights the considerable opportunities for scaling up this activity. Stakeholder engagement has further underscored the need to develop regulatory frameworks to guide this emerging land use. Establishing clear regulations will be essential to ensure sustainable harvesting practices and the production of high-quality, environmentally responsible end products.

### Supporting information

Potential *Sphagnum* moss harvesting sites: free downloadable dataset on the potential harvesting sites for *Sphagnum* moss biomass. The data includes the delineation of the object and a natural state class. https://hakku.gtk.fi/en/locations?id=505.

## Supplementary information

Supplementary Material

## Data Availability

No datasets were generated or analyzed during the current study.

## References

[CR1] Aapala K, Kartano L, Määttänen A-M, Alanen A (2021) Soidensuojelun täydennysehdotus—Tilannekatsaus 2015–2020. Complementary mire conservation programme Situation report 2015-2020. Publ Minist Environ 2021:16.

[CR2] Ågren AM, Anderson O, Lidberg W, Öquist M, Hasselquist EM (2024) Ditches show systematic impacts on soil and vegetation properties across the Swedish forest landscape. Ecol Manag 555:121707. 10.1016/j.foreco.2024.121707

[CR3] Alanen A, Aapala K (2015) Soidensuojelutyöryhmän ehdotus soidensuojelun täydentämiseksi. Proposal of the Mire Conservation Group for supplemental mire conservation. Rep Minist Environ 26/2015:1–175.

[CR4] Blok C, Eveleens B, van Winkel A (2021) Growing media for food and quality of life in the period 2020-2050. Acta Hortic 1305:341–356. 10.17660/ActaHortic.2021.1305.46

[CR5] Van Breemen N (1995) How *Sphagnum* bogs down other plants. Trends Ecol Evol 10(7):270–275. 10.1016/0169-5347(95)90007-1 21237035 10.1016/0169-5347(95)90007-1

[CR6] Britschgi R, Rintala J, Puharinen S-T (2018) Pohjavesialueet - Opas määrittämiseen, luokitukseen ja suojelusuunnitelmien laadintaan. Groundwater areas - a guide for their designation and classification and preparation of protection plans. Environ Adm Guide 3/2018:1796–1653.

[CR7] Clymo RS, Hayward PM (1982) The ecology of *Sphagnum*. In A. J. E. Smith (Ed.), Bryophyte ecology (pp. 229–289). Chapman and Hall. 10.1007/978-94-009-5891-3_8

[CR8] Finnish Environmental Institute (2023a) Syke’s metadata portal. Groundwater bodies. https://ckan.ymparisto.fi/dataset/pohjavesialueet (accessed 27 January 2023)

[CR11] Finnish Environmental Institute (2023b) Syke’s metadata portal. The nature protected areas and wilderness reserves dataset. https://ckan.ymparisto.fi/dataset/luonnonsuojelu-ja-eramaa-alueet (accessed 15 March 2023)

[CR10] Finnish Environmental Institute (2023c) Syke’s metadata portal. The Natura 2000 dataset. https://ckan.ymparisto.fi/fi/dataset/natura2000-alueet (accessed 21 March 2023)

[CR9] Finnish Environmental Institute (2023d) Syke’s metadata portal. Soidensuojelun täydennysehdotus ja valtionmaan toteutuneet kohteet. Complementary mire conservation programme https://ckan.ymparisto.fi/dataset/soidensuojelun-taydennysehdotus-ja-valtionmaan-toteutuneet-kohteet (accessed 15 March 2023)

[CR12] Finnish Government [Valtioneuvosto] (2012) Valtioneuvoston periaatepäätös soiden ja turvemaiden kestävästä ja vastuullisesta käytöstä ja suojelusta. Decision-in-Principle/ Government resolution on “Sustainable and Responsible Use and Protection of Peatlands” (in Finnish) https://mmm.fi/documents/1410837/1516663/mmm-119690-v5-suostrategia_valtioneuvoston_periaatepaatos_v4/005425e8-e3c4-497d-8cff-26f343896c37

[CR13] Finnish Heritage Agency (2023) Spatial data sets on the cultural environment. https://www.museovirasto.fi/en/services-and-guidelines/data-systems/kulttuuriympaeristoen-tietojaerjestelmae/kulttuuriympaeristoen-paikkatietoaineistot (accessed 1 February 2023)

[CR14] Finnish Transport Infrastructure Agency (2023) Digiroad. https://ava.vaylapilvi.fi/ava/Tie/Digiroad (accessed 29 August 2023)

[CR15] Forest Centre (2022) https://www.metsakeskus.fi/fi/avoin-metsa-ja-luontotieto/luontotietoaineistot/erityisen-tarkeat-elinymparistot (accessed 26 October 2022)

[CR16] Gao Q, Yang G, Xue J, Huang X (2023) Characteristics of n-alkyl lipids in cultivated *Sphagnum* and their preservation potential in the topsoil of *Sphagnum* farmlands in southwest China. Org Geochem 185:104677. 10.1016/j.orggeochem.2023.104677

[CR17] Gaudig G, Krebs M, Prager A, Wichmann S, Barney M, Caporn SJM, Emmel M, Fritz C, Graf M, Grobe A, Gutierrez Pacheco S, Hogue-Hugron S, Holzträger S, Irrgang S, Kämäräinen A, Karofeld E, Koch G, Koebbing JF, Kumar S, Matchutadze I, Oberpaur C, Oestmann J, Raabe P, Rammes D, Rochefort L, Schmilewksi G, Sendžikaitė J, Smolders A, St-Hilaire B, van de Riet B, Wright B, Wright N, Zoch L, Joosten H (2018) *Sphagnum* farming from species selection to the production of growing media: A review. Mires Peat 20:1–30. 10.19189/MaP.2018.OMB.340

[CR18] Geological Survey of Finland (2024) Spatial dataset on *Sphagnum* moss harvesting sites. https://hakku.gtk.fi/fi/locations?id=232 (accessed 1 September 2024)

[CR19] Geological Survey of Finland (2025) Sphagnum moss harvesting sites (freely available spatial data product) https://hakku.gtk.fi/en/locations?id=232 (Site accessed 2.6.2025)

[CR20] Guêné-Nanchen M, St-Hilaire B (2022) *Sphagnum* Farming in Canada: State of Knowledge. 60. CSPMA and APTHQ. Québec, Quebec. https://tourbehorticole.com/wp-content/uploads/2023/01/APTHQ-Guide_Culture_sphaignes_ANG_web.pdf

[CR21] Heiskanen J (2021) Kasvuturpeen tulevaisuuden näkymät ja vaihtoehdot (The future and alternatives for horticultural peat). Natural Resources Institute Finland. [Webinar] (in Finnish) https://www.slideshare.net/slideshow/kasvuturpeen-tulevaisuuden-nkymt-ja-vaihtoehdot-juha-heiskanen-luke/242102610

[CR22] Hermanns T, Helming K, König HJ, Schmidt K, Li Q, Faust H (2017) Sustainability impact assessment of peatland-use scenarios: confronting land use supply with demand. Ecosyst Serv 26:365–376. 10.1016/j.ecoser.2017.02.002

[CR23] Herranen N (2018) Luonnonarvojen huomioon ottaminen turvetuotannon sijoittamisessa (”Ecological values and peat production”, abstract available in English). Ympäristöjuridiikka 2–3/2018:9–41.

[CR24] Hirschler O, Osterburg B, Weimar H Glasenapp S, Ohmes MF (2022) Peat replacement in horticultural growing media: Availability of bio-based alternative materials. Braunschweig: Johann Heinrich von Thünen-Institut, 64 p. Thünen Working Paper 190, 10.3220/WP1648727744000

[CR25] Hoshi Y (2017) Sphagnum growth in floating cultures: Effect of planting design. Mires Peat 20:1–10. 10.19189/MaP.2017.OMB.294

[CR26] Karjalainen SK, Anttila J, Maanavilja L, Hamedianfar A, Laine AM (2025) Carbon dioxide and methane gas exchange following sphagnum moss harvesting in boreal peatland. J Environ Manag 373:123357. 10.1016/j.jenvman.2024.123357 10.1016/j.jenvman.2024.123357 39603099

[CR27] Kokkonen NAK, Laine AM, Laine J, Vasander H, Kurki K, Gong J, Tuittila E-S (2019) Responses of peatland vegetation to 15-year water level drawdown as mediated by fertility level. J Veg Sci 30(6):1206–1216. 10.1111/jvs.12794

[CR28] Laakso T, Heinilä A (2023) Rahkasammalen korjuun ohjauskeinot ja niiden kehittäminen (Assessment of the current legal framework from the point of view of *Sphagnum* moss harvesting and the need for regulatory action, abstract available in English). Publ Univ East Finl-Rep Stud Soc Sci Bus Stud 20:ISBN 978-952-61-4908-0

[CR29] Laatikainen A, Kolari THM, Tahvanainen T (2025) Sphagnum moss layer growth after restoration of forestry-drained peatlands in Finland. Restor Ecol 33(4):e70008. 10.1111/rec.70008

[CR30] Laiho R, Tuominen S, Kojola S, Penttilä T, Saarinen M, Ihalainen A (2016) Heikkotuottoiset ojitetut suometsät - missä ja paljonko niitä on? (Unproductive forestry-drained peatlands - location and total area). Metsätieteen aikakauskirja 2:73–93

[CR31] Laine-Petäjäkangas A, Anttila J, Maanavilja L, Uusheimo S, Vuorenmaa J, Myllyviita T, Lampela M, Karvonen J, Hamedianfar A, Allonen O, Grönroos J, Lehtoranta S, Ikkala L, Karjalainen S, Kivilompolo J, Silvan N, Sutinen H, Turunen J (2024) Rahkasammalesta ilmastoviisas kasvualusta – mahdollisuudet kokonaiskestävään korjuuseen (RahKoo) -hankkeen loppuraportti (“Climate-Smart Growing Medium from Sphagnum Moss – Opportunities for Sustainable Harvesting (RahKoo)”-project report). Geological Survey of Finland, Open File Research Report 80/2024. https://hakku.gtk.fi/fi/publications?id=100112

[CR32] Laine J, Vasander H, Laiho R (1995) Long-term effects of water level drawdown on the vegetation of drained pine mires in southern Finland. J Appl Ecol 32(4):785–802. 10.2307/2404818

[CR33] Laine J, Vasander H, Hotanen J-P, Nousiainen H, Saarinen M, Penttilä T (2018) Suotyypit ja turvekankaat – kasvupaikkaopas (Forest peatland and heathy peatland types - classification guide). 160. Metsäkustannus Oy, Helsinki, Finland ISBN 978-952-338-036-3 (in Finnish)

[CR35] Loisel J, Yu Z, Beilman DW, Camill P, Alm J, Amesbury MJ, Anderson D, Andersson S, Bochicchio C, Barber K, Belyea LR, Bunbury J, Chambers FM, Charman DJ, De Vleeschouwer F, Fiałkiewicz-Kozieł B, Finkelstein SA, Gałka M, Garneau M, Zhou W (2014) A database and synthesis of northern peatland soil properties and Holocene carbon and nitrogen accumulation. Holocene 24(9):1028–1042. 10.1177/0959683614538073

[CR36] Natural Resources Institute Finland (2021) Statistics database. Production, consumption and foreign trade of peat 1970-2021 https://statdb.luke.fi/PxWeb/pxweb/en/LUKE/LUKE__04%20Metsa__08%20Muut__Energia/?rxid=001bc7da-70f4-47c4-a6c2-c9100d8b50db Site accessed 30.5.2025

[CR37] Mäkisara K, Katila M, Peräsaari J (2022) The Multi-Source National Forest Inventory of Finland - methods and results 2017 and 2019. Nat Resour Bioecon Stud 90/2022. Helsinki. http://urn.fi/URN:ISBN:978-952-380-538-5

[CR38] McKeon-Bennett MMP, Hodkinson TR (2021) *Sphagnum* moss as a novel growth medium in sustainable indoor agriculture systems. Curr Opin Environ Sci Health 22:100269. 10.1016/j.coesh.2021.100269

[CR39] Middleton M, Laatikainen M, Kivilompolo J, Harju A, Lerssi J, Valkama M, Pitkänen T, Pohjankukka J, Balazs A, Tuominen S, Zelioli L, Farahnakian F, Nevalainen P, Heikkonen J (2023) Technical description for the peatland site type data of Finland. GTK Open File Work Report https://tupa.gtk.fi/raportti/arkisto/73_2023.pdf

[CR40] Ministry of the Environment (2015) Suot ja turvemaat maakuntakaavoituksessa (Peatlands in regional land use planning). Suomen ympäristö 7 | 2015. pp. 112. Ministry of the Environment. Helsinki. ISSN 1796-1637 (in Finnish) http://urn.fi/URN:ISBN:978-952-11-4460-8

[CR41] Ministry of the Environment (2022) Rahkasammalen korjuun ympäristövaikutukset ─ yhteistyöryhmän loppuraportti (Environmental impact of *Sphagnum* moss harvesting ─ final report of the committee addressing *Sphagnum* moss harvesting) (in Finnish) https://api.hankeikkuna.fi/asiakirjat/bdefe868-d888-4b4f-8c86-e8820fa18cab/12e369cf-4fb0-47cb-aada-31b87f4dba7c/RAPORTTI_20220221110514.PDF

[CR42] Müller R, Glatzel S (2021) *Sphagnum* farming substrate is a competitive alternative to traditional horticultural substrates for achieving desired hydro-physical properties. Mires Peat 27(21):1–12. 10.19189/MaP.2021.OMB.StA.2157

[CR43] Myllyviita T, Karjalainen S, Anttila J, Grönroos J, Turunen J, Lehtoranta S, Laine AM (preprint 2025) Life Cycle Analysis of Greenhouse Gas Emissions of Sphagnum Moss Harvesting and Use: Comparison with Horticultural Peat” for Consideration by Ecological Engineering (SSRN Scholarly Paper 5114669). Social Science Research Network. 10.2139/ssrn.5114669

[CR44] National Land Survey of Finland (2023) The Topographic database. https://www.maanmittauslaitos.fi/en/geopackage (accessed 29 August 2023)

[CR45] Natural Resources Institute Finland (2023) File service for publicly available data. https://kartta.luke.fi/index-en.html (accessed 21 March 2023)

[CR46] Nielsen M, Bryndum N, Bedsted B (2017) Organising stakeholder workshops in research and innovation – between theory and practice. J Public Delib 13(2). 10.16997/jdd.285

[CR47] Ørngreen R, Levinsen KT (2017) Workshops as a Research Methodology. Electron J E-Learn 15(1):70–81

[CR48] Pouliot R, Hugron S, Rochefort L (2015) *Sphagnum* farming: A long-term study on producing peat moss biomass sustainably. Ecol Eng 74:135–147. 10.1016/j.ecoleng.2014.10.007

[CR49] Le Quéré C, Andrew RM, Friedlingstein P, Sitch S, Hauck J, Pongratz J, Pickers PA, Korsbakken JI, Peters GP, Canadell JG, Arneth A, Arora VK, Barbero L, Bastos A, Bopp L, Chevallier F, Chini LP, Ciais P, Doney SC, Gkritzalis T, Goll DS, Harris I, Haverd V, Hoffman FM, Hoppema M, Houghton RA, Hurtt G, Ilyina T, Jain AK, Johannessen T, Jones CD, Kato E, Keeling RF, Goldewijk KK, Landschützer P, Lefèvre N, Lienert S, Liu Z, Lombardozzi D, Metzl N, Munro DR, Nabel JEMS, Nakaoka S, Neill C, Olsen A, Ono T, Patra P, Peregon A, Peters W, Peylin P, Pfeil B, Pierrot D, Poulter B, Rehder G, Resplandy L, Robertson E, Rocher M, Rödenbeck C, Schuster U, Schwinger J, Séférian R, Skjelvan I, Steinhoff T, Sutton A, Tans PP, Tian H, Tilbrook B, Tubiello FN, van der Laan-Luijkx IT, van der Werf GR, Viovy N, Walker AP, Wiltshire AJ, Wright R, Zaehle S, Zheng B (2018) Global Carbon Budget 2018. Earth Syst Sci Data 10:2141–2194. 10.5194/essd-10-2141-2018.

[CR50] Quinty F, Rochefort L (2003) Peatland Restoration Guide (second ed.), Canadian Sphagnum Peat Moss Association and New Brunswick Department of Natural Resources and Energy, Québec, Québec. ISBN 0-9733016-0-0 https://www.gret-perg.ulaval.ca/fileadmin/Fichiers/centre_recherche/Peatland_Restoration_guide_2ndEd.pdf

[CR51] Reed MS, Vella S, Challies E, de Vente J, Frewer L, Hohenwallner-Ries D, Huber T, Neumann RK, Oughton EA, Sidoli del Ceno J, van Delden H (2018) A theory of participation: what makes stakeholder and public engagement in environmental management work?. Restor Ecol 26:S7–S17. 10.1111/rec.12541

[CR52] Sallinen A, Tuominen S, Kumpula T, Tahvanainen T (2019) Undrained peatland areas disturbed by surrounding drainage: a large scale GIS analysis in Finland with a special focus on aapa mires. Mires Peat ume 24:1–22. 10.19189/MaP.2018.AJB.391

[CR53] Silvan N, Sarkkola S, Laiho R (2019) Rahkasammalbiomassa ja sen korjuuseen soveltuvat suot Suomessa (Summary available in English: Peatlands suitable for harvesting of renewable *Sphagnum* moss biomass in Finland). Suo 70(2–3):41–53

[CR54] SLL (2020a) Rahkasammalen nosto uhkaa luonnontilaisia soita – toiminta saatava luvanvaraiseksi (*Sphagnum* harvesting threatens pristine peatlands – activity needs to be licensed) (in Finnish) https://www.sll.fi/2020/10/20/rahkasammalen-nosto-uhkaa-luonnontilaisia-soita-toiminta-saatava-luvanvaraiseksi/

[CR55] SLL (2020b) Esitys ympäristöministeriölle. Esitys: Rahkasammalen nosto luvanvaraiseksi (Proposal for the Ministry of Environment: *Sphagnum* harvesting to become licensed) (in Finnish) https://www.sll.fi/app/uploads/sites/10/2020/10/Esitys_YMlle_20102020_Rahkasammalen_nosto_luvanvaraiseksi-1.pdf

[CR56] SLL (2022) Suomen luonnonsuojeluliitto vaatii: rahkasammalen korjuuta ei saa tehdä ennen kuin lainsäädäntö on ajan tasalla (The Finnish Association for Nature Conservation requires: *Sphagnum* harvesting must not be done until the legislation is up-to-date). Newsletter (in Finnish) https://www.sll.fi/2022/02/04/suomen-luonnonsuojeluliitto-vaatii-rahkasammalen-korjuuta-ei-saa-tehda-ennen-kuin-lainsaadanto-on-ajan-tasalla/

[CR58] Taskila S, Särkelä R, Tanskanen J (2016) Valuable applications for peat moss. Biomass–Conv Bioref 6:115–126. 10.1007/s13399-015-0169-3

[CR59] Toivonen T, Herranen T, Kivilompolo J, Kujala H, Laatikainen M, Suomi T, Turunen J, Valo O, Vähäkuopus T (2022) GTK:n tutkimien soiden tutkimustilanne ja luonnontilaisuusluokitukset maakunnittain. Summary available in English: Peatland research by the Geological Survey of Finland and classification of the natural state of peatlands. Geological Survey of Finland, Open File Research Report 40/2022 (in Finnish) https://tupa.gtk.fi/raportti/arkisto/40_2022.pdf

[CR60] Turunen J, Valpola S (2020) The influence of anthropogenic land use on Finnish peatland area and carbon stores 1950–2015. Mires Peat 26(26):1–27. 10.19189/MaP.2019.GDC.StA.1870

[CR61] Väyrynen T, Aaltonen R, Haavikko H, Juntunen M, Kalliokoski K, Niskala A-L, Tukiainen O (2008) Turvetuotannon ympäristönsuojeluopas (Environmental protection guide for peat production). Pohjois-Pohjanmaan ympäristökeskus. ISSN 1796-167X (in Finnish) http://hdl.handle.net/10138/38820

[CR62] Water Act VL 587/2011 https://www.finlex.fi/fi/laki/ajantasa/2011/20110587

