# Supplementary material for: Developing Criteria for an Emerging Land Use - Sphagnum Moss Harvesting - Through Stakeholder Engagement and Consequent Potential Sphagnum Harvesting Area in Finland
Source: Environ Manage. 2025 Sep 30;75(12):3586–600. doi: 10.1007/s00267-025-02279-y (PMC12575481; doi:10.1007/s00267-025-02279-y)
Supplement: Supplementary file 1 — Supplementary Material [file 267_2025_2279_MOESM1_ESM.docx]

SUPPLEMENTARY MATERIAL

**Table S1. a)** Potential *Sphagnum* harvesting area resulting from spatial analysis with 50 m distance to the nearest ditch, and its consecutive effect on the Yield and Production cost categories. The effect of changes in thresholds in Yield and Production cost categories are presented in **b)** and **c).** *The total peatland area (6 183 400 ha) contains also the natural states 3, 4 and 5 (854 268 ha, 798 749 ha and 931 415 ha, respectively).

c)

b)

a)
